# Supplementary material for: A large multiethnic GWAS meta-analysis of cataract identifies new risk loci and sex-specific effects
Source: Nat Commun. 2021 Jun 14;12:3595. doi: 10.1038/s41467-021-23873-8 (PMC8203611; doi:10.1038/s41467-021-23873-8)

Choquet et al. "A large multiethnic GWAS meta-analysis of cataract identifies new risk loci and sex-specific effects"

Supplementary Information

Supplementary Figure 1. Flowchart of the study design.

Supplementary Figure 2. QQ plot and genomic inflation factors (λ) observed for the combined (GERA+UKB) multiethnic meta-analysis of cataract.

Supplementary Figure 3. Correlation of effect sizes for cataract between GERA and UKB cohorts for the lead 54 cataract-associated lead SNPs identified in the combined (GERA+UKB) multiethnic meta-analysis.

Supplementary Figure 4. Correlation of effect sizes for cataract between the meta-analysis (GERA+UKB) results and 23andMe dataset for the lead 54 cataract-associated lead SNPs identified in the combined (GERA+UKB) multiethnic meta-analysis.

Supplementary Figure 5. Manhattan plots of the GWAS of cataract (GERA + UKB) stratified by ethnic groups. **a.** European ancestry; **b.** East Asian ancestry; and **c.** African ancestry.

Supplementary Figure 6. LocusZoom plots of regions identified in the combined (GERA + UKB) meta-analysis of cataract stratified by ethnicity. We identified three additional novel regions specific to individuals of European ancestry: **a.** *EPHA4*, **b.** *CD83-JARID2*, and **c.** *EXOC3L2*.

Supplementary Figure 7. LocusZoom plots of regions identified in the sex-specific (GERA+UKB) analyses that show differential association with cataract across women and men. The following regions were significant in women ($P<5x10^{-8}$) but not significant ($P>0.05$) in men: **a.** *GSTM2*, **b.** *DNMBP-CPN1*, and **c.** *CASP7*; the following regions were genome-wide significant in men but not in women: **d.** *QKI*, **e.** *SEMA4D*, **f.** *RBFOX1*, and **g.** *JAG1*.

Supplementary Figure 8. Correlation of effect sizes for cataract between women and men for the lead 54 SNPs identified in the combined (GERA+UKB) GWAS multiethnic analysis and for the additional SNPs identified in the sex-specific analyses.

Supplementary Figure 9. Enriched expression of candidate genes in mouse lens.

Supplementary Figure 10. Expression of candidate genes in lenses of gene perturbation mouse models with lens defects/cataract.

Supplementary Figure 11. RT-PCR based validation of candidate gene expression in the lens

Supplementary Figure 1. Flowchart of the study design

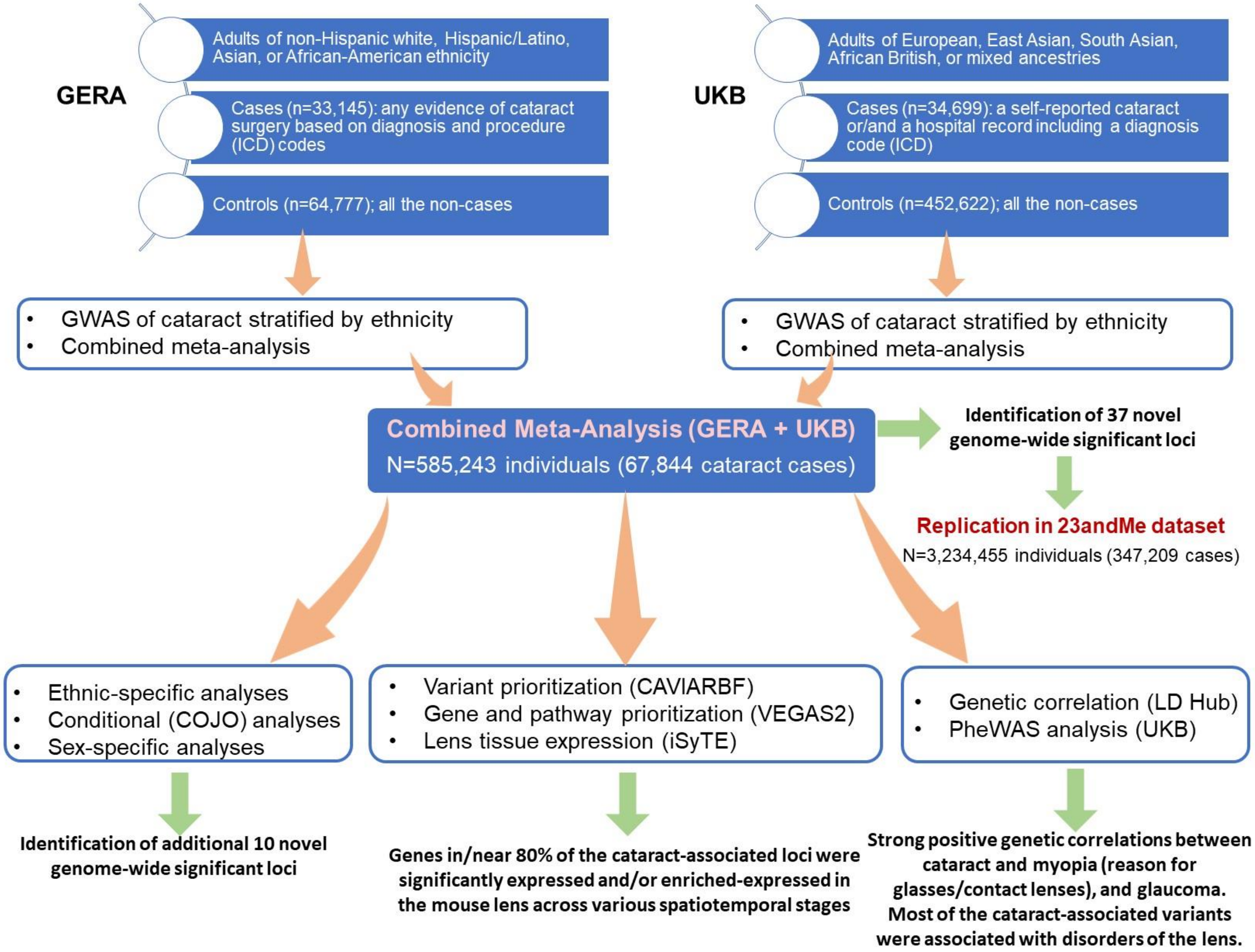

Supplementary Figure 2. QQ plot and genomic inflation factors (λ) observed for the combined (GERA+UKB) multiethnic meta-analysis of cataract

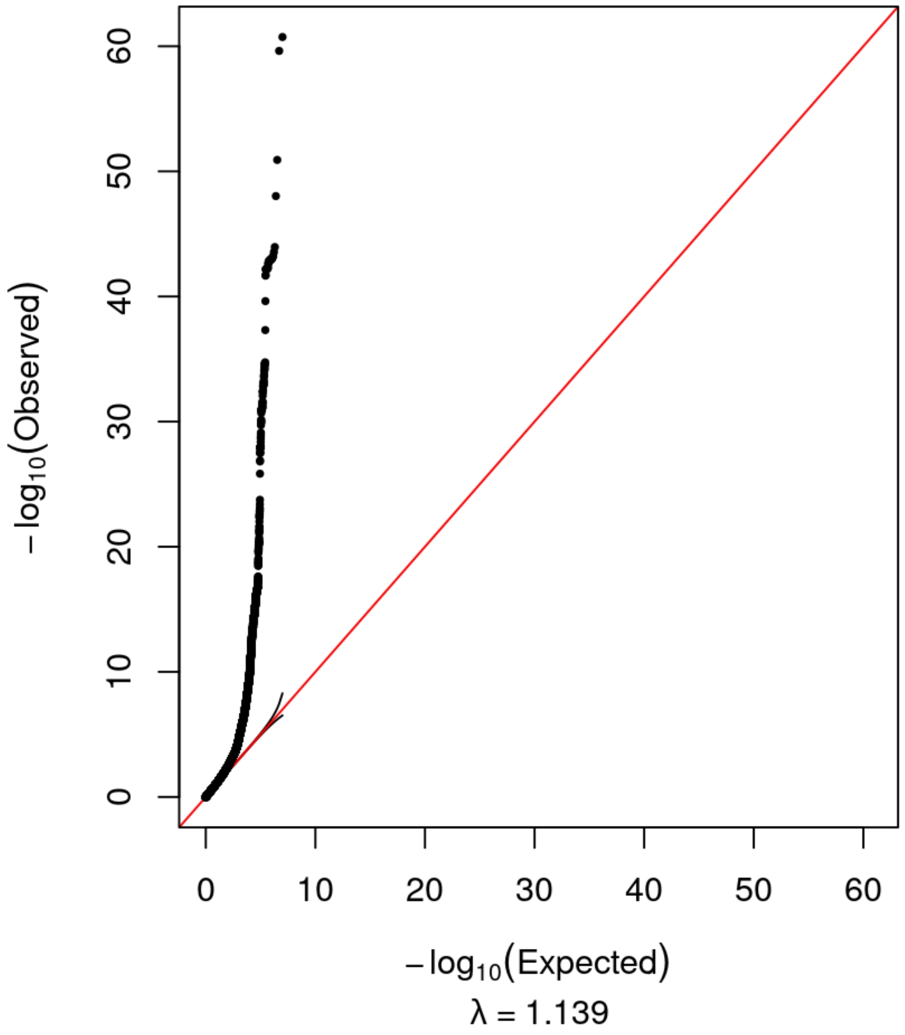

Supplementary Figure 3. Correlation of effect sizes for cataract between GERA and UKB cohorts for the lead 54 cataract-associated lead SNPs identified in the combined (GERA+UKB) multiethnic meta-analysis. The effect sizes were compared using a correlation test, two-sided; *P*-value for the correlation test is 4.1×10^{-12}.

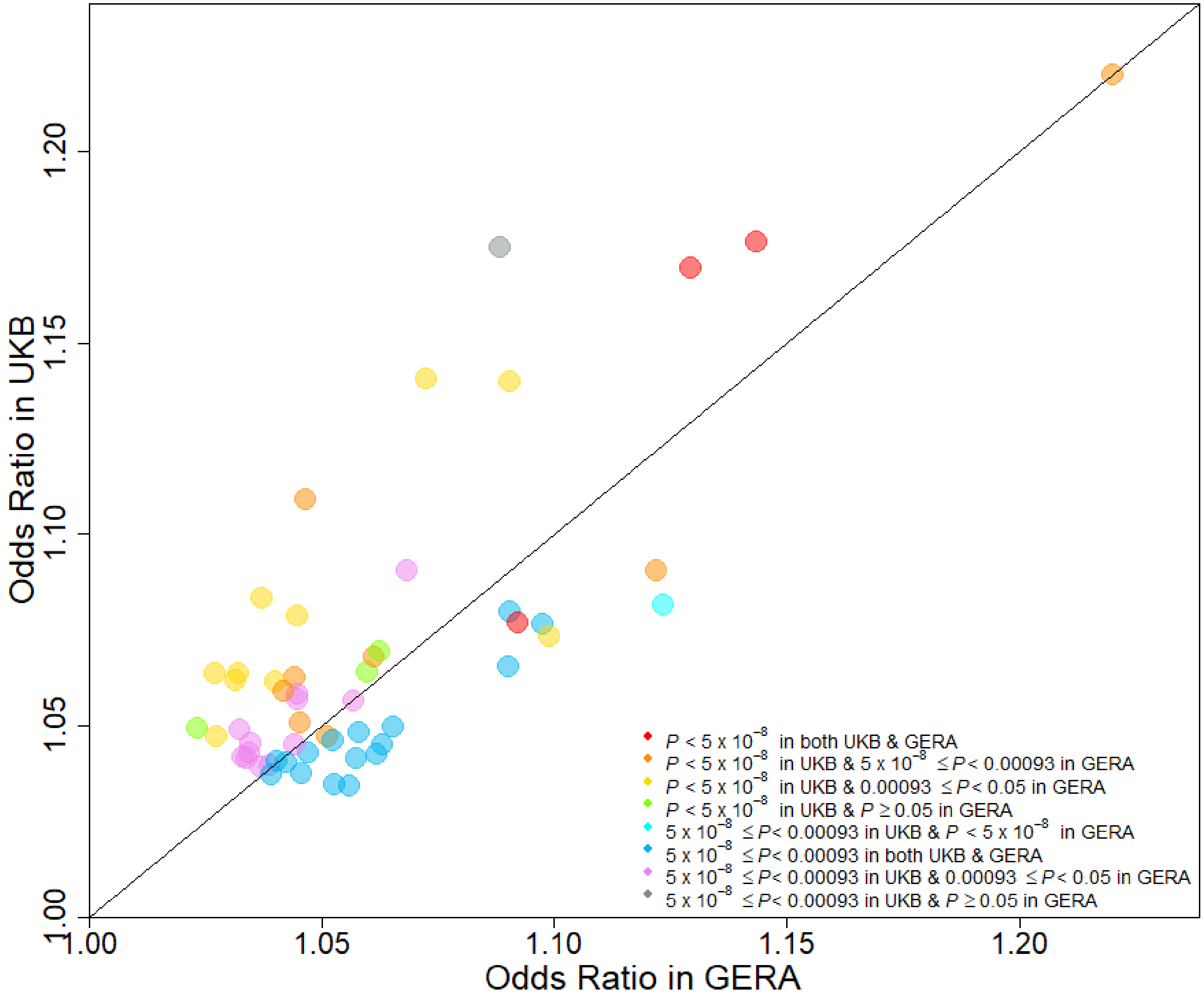

Supplementary Figure 4. Correlation of effect sizes for cataract between the meta-analysis (GERA+UKB) results and 23andMe dataset for the lead 54 cataract-associated lead SNPs identified in the combined (GERA+UKB) multiethnic meta-analysis. The effect sizes were compared using a correlation test, two-sided; *P*-value for the correlation test is 1.0×10^{-14}.

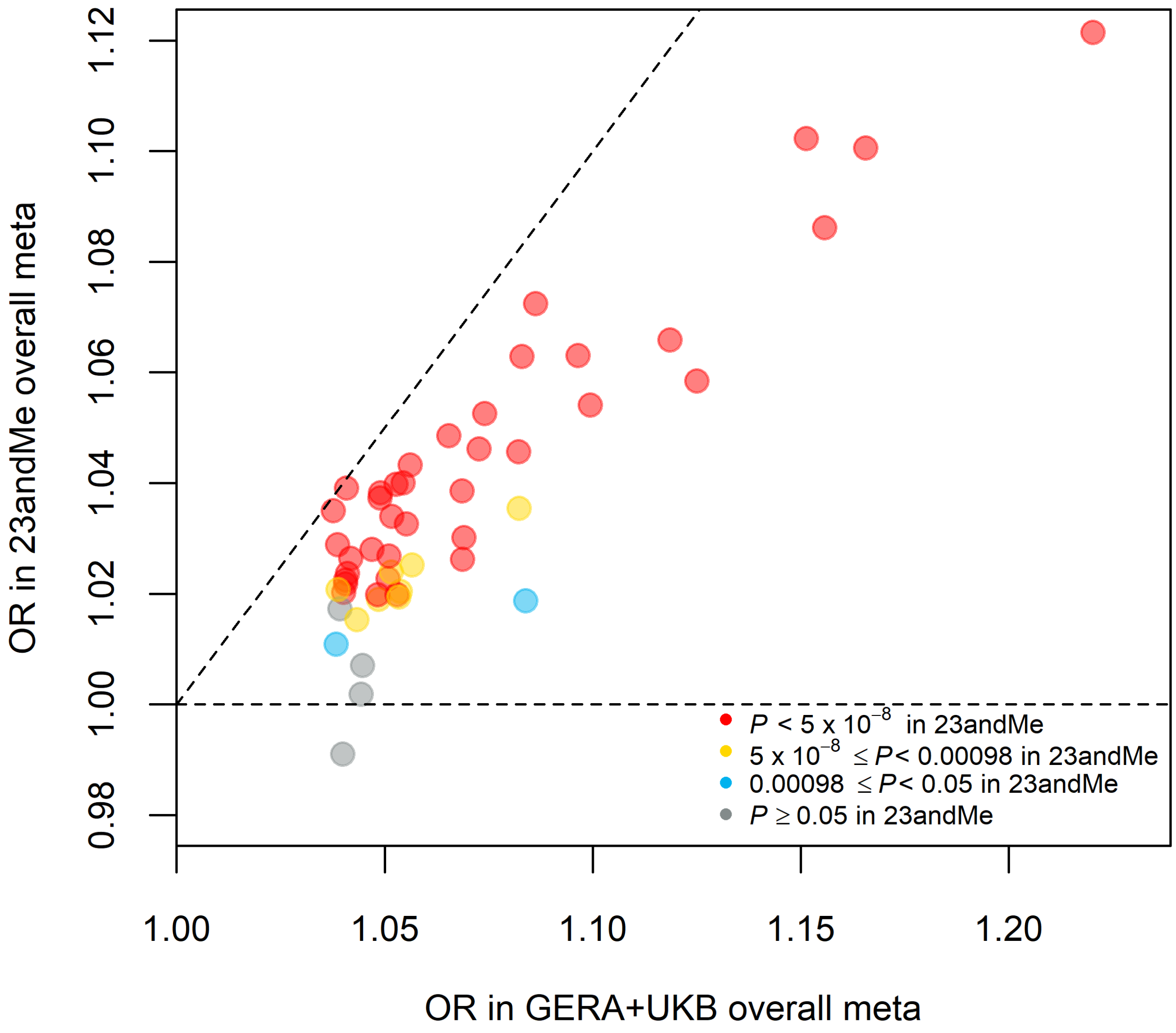

Supplementary Figure 5. Manhattan plots of the GWAS of cataract (GERA + UKB) stratified by ethnic groups. **a.** European ancestry; **b.** East Asian ancestry; and **c.** African ancestry

a.

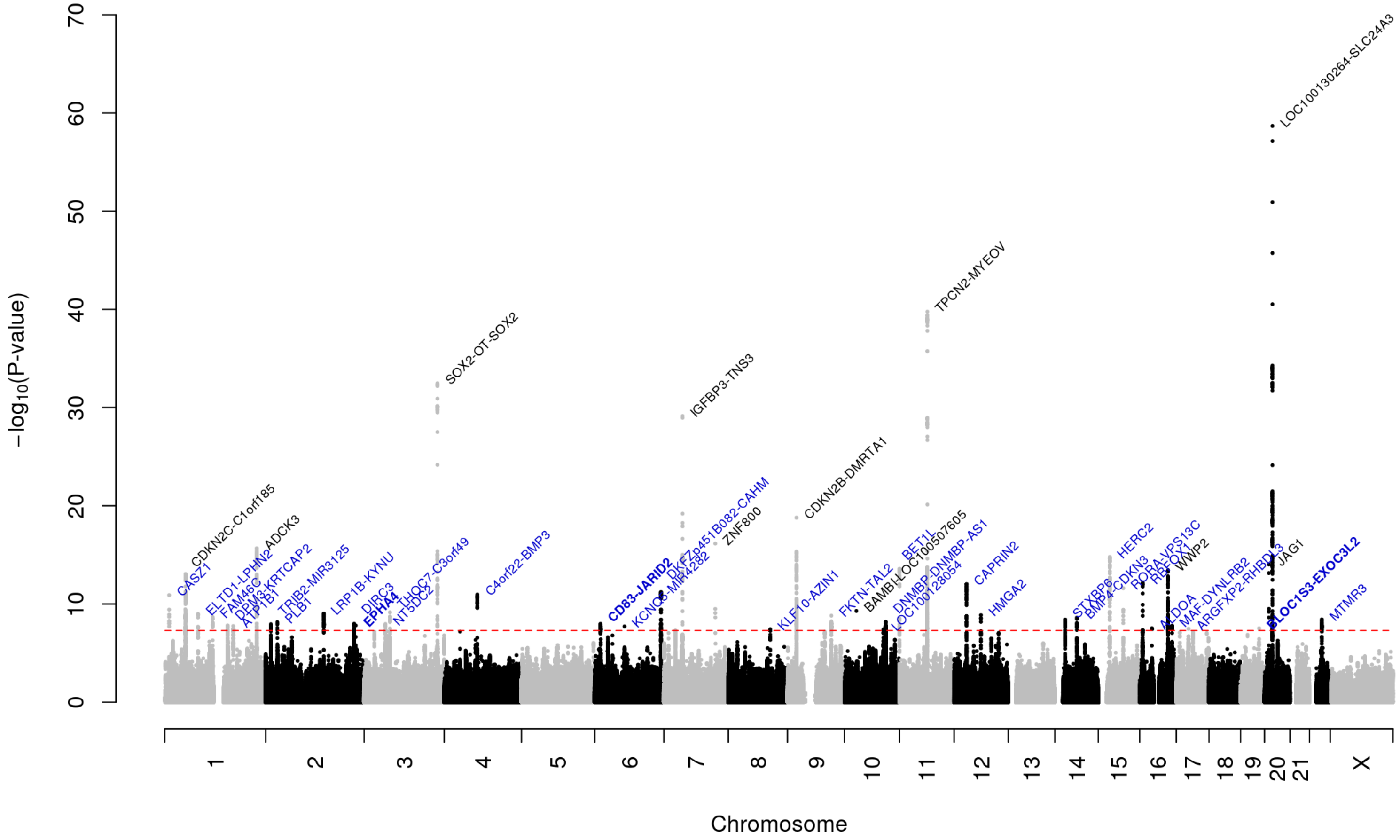

Locus names in blue are for the novel loci and the ones in dark are for the previously reported ones. Locus names in bold are additional novel (compared to the multiethnic meta-analysis (GERA+UKB) results).

b.

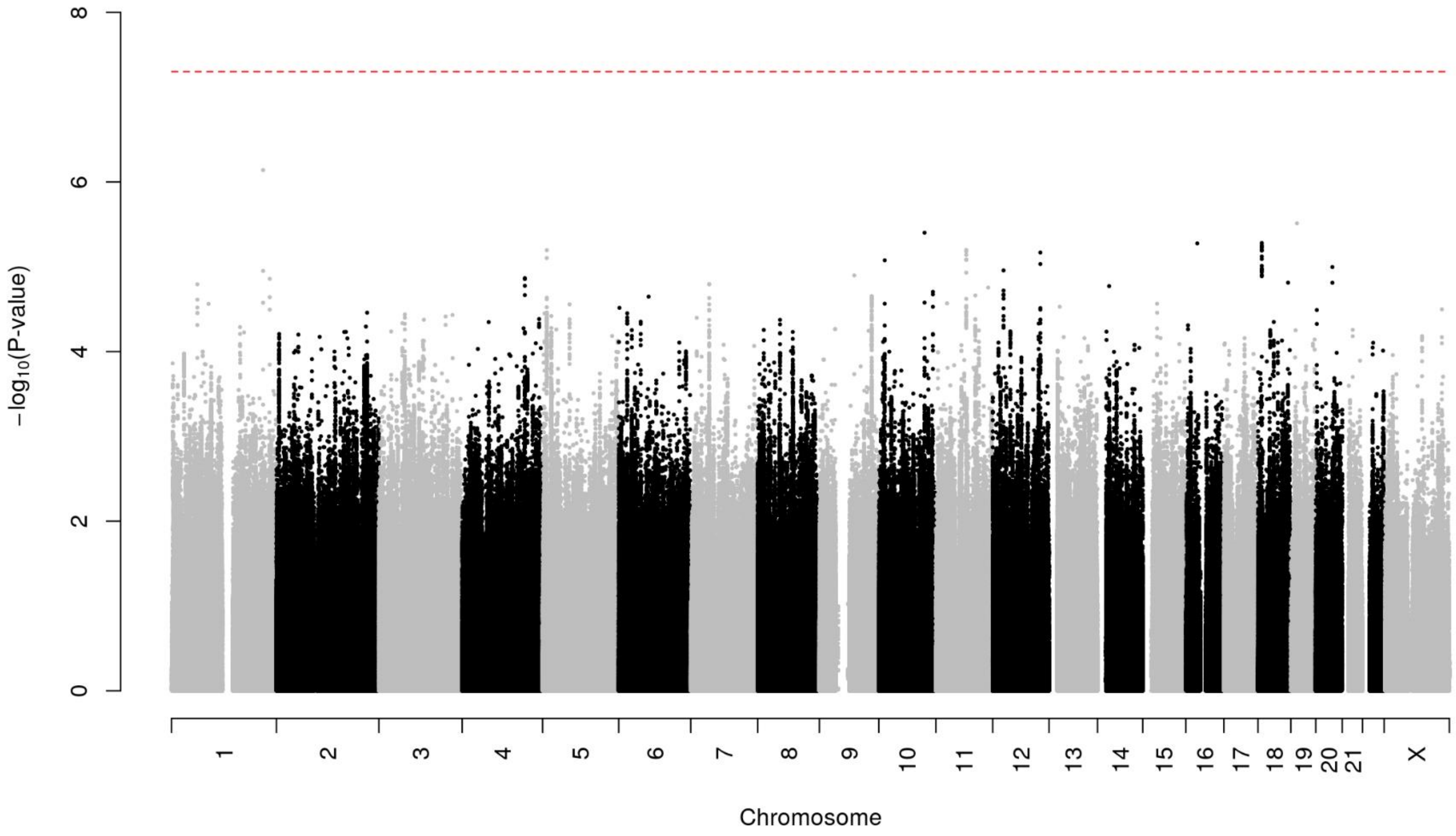

8
6
4
2
0
−log10(P-value)
1
2
3
4
5
6
7
8
9
10
11
12
13
14
15
16
17
18
19
20
21
X
Chromosome

c.

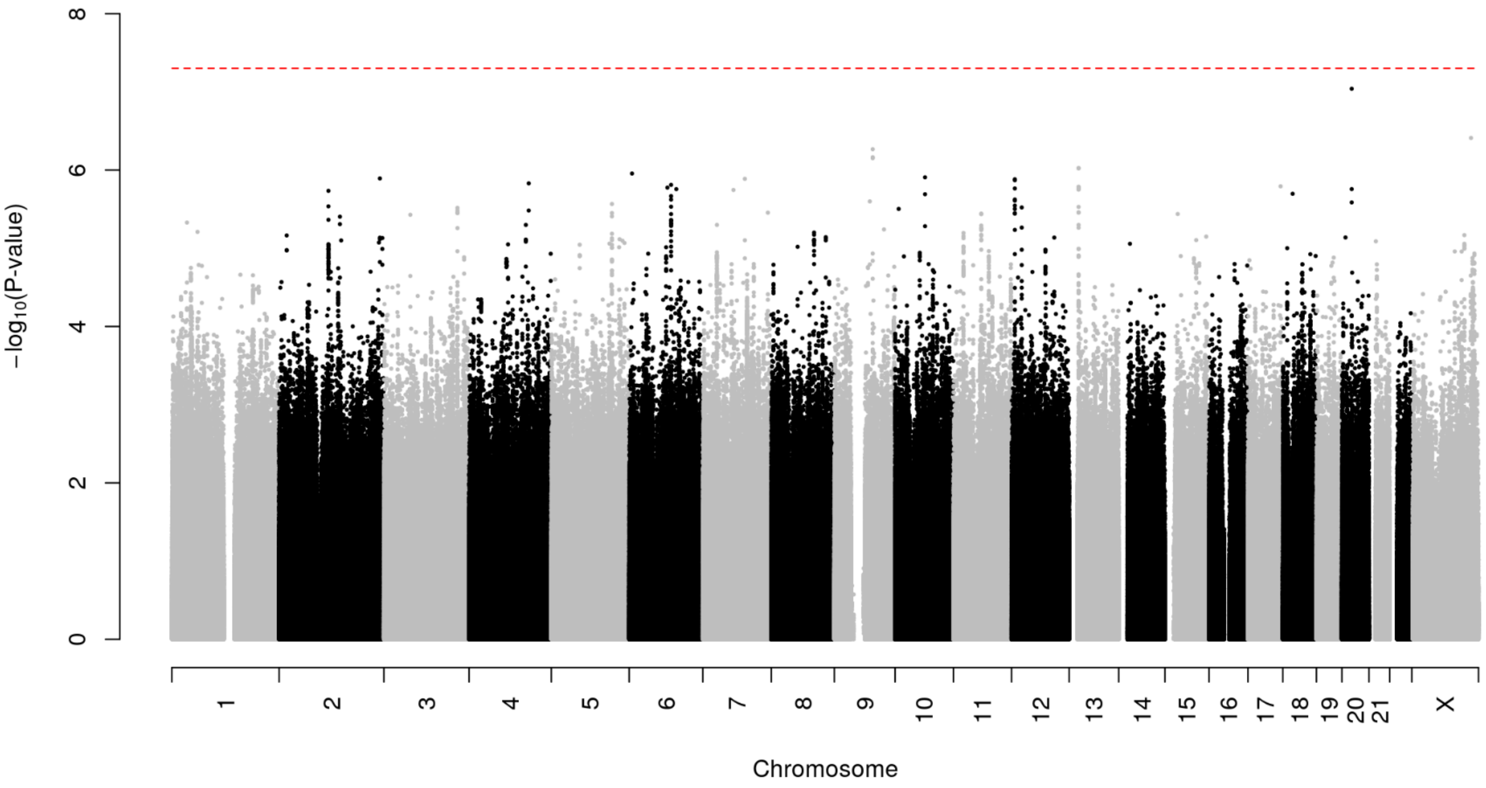

8
6
4
2
0
−log10(P-value)
1
2
3
4
5
6
7
8
9
10
11
12
13
14
15
16
17
18
19
20
21
X
Chromosome

Supplementary Figure 6. LocusZoom plots of regions identified in the combined (GERA + UKB) meta-analysis of cataract stratified by ethnicity. We identified three additional novel regions specific to individuals of European ancestry: **a.** *EPHA4*, **b.** *CD83-JARID2*, and **c.** *EXOC3L2*

a.

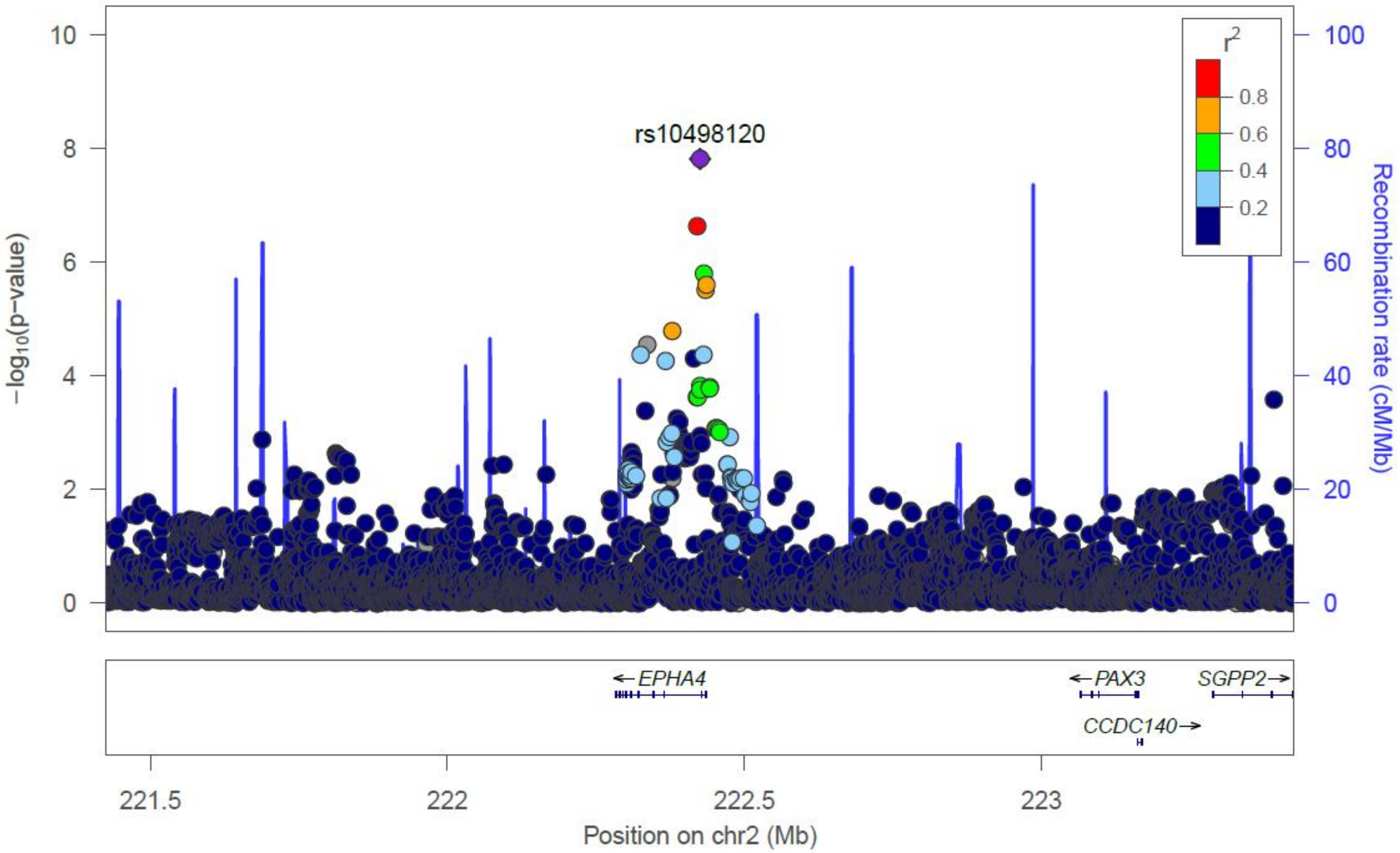

b.

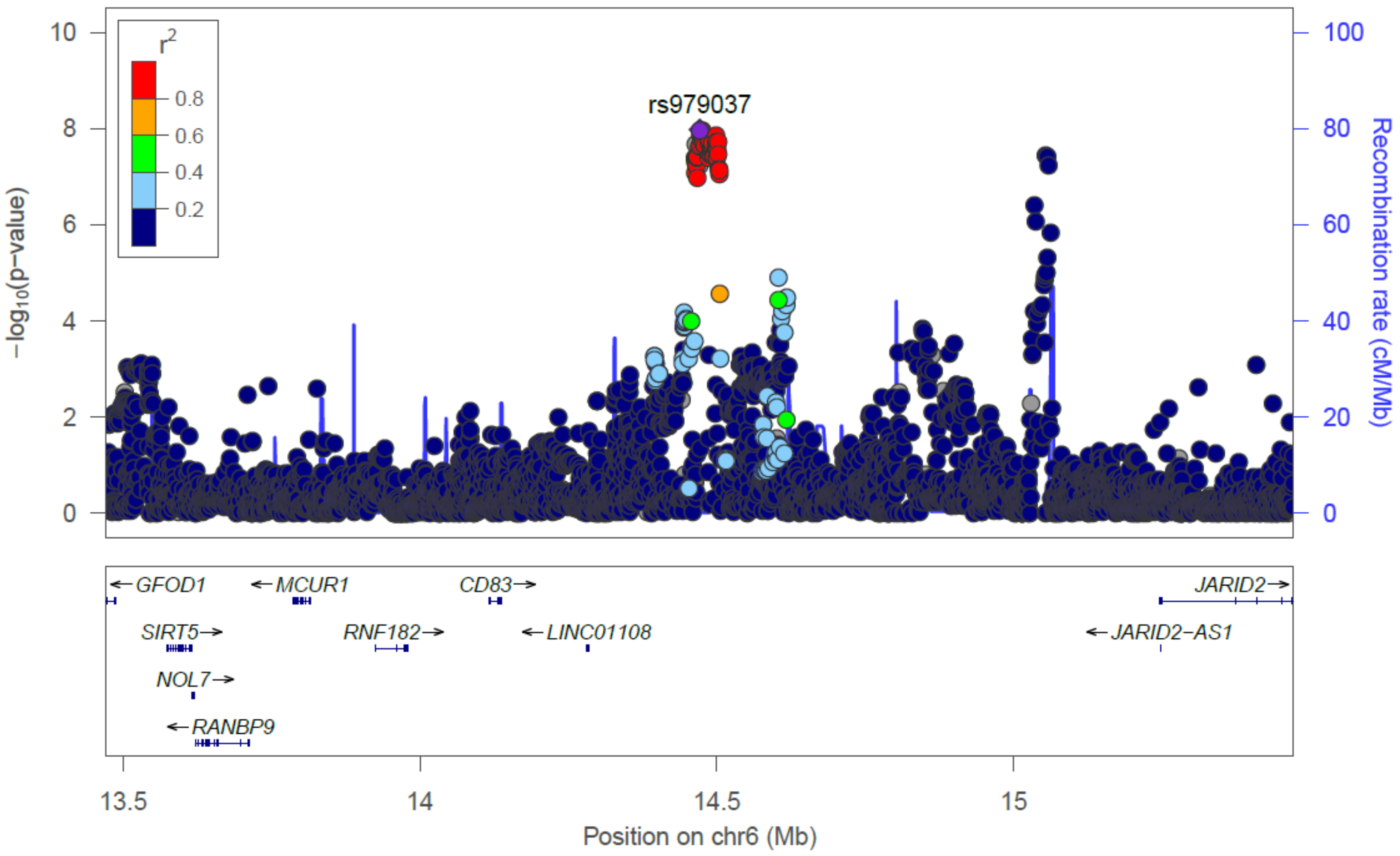

r2
0.8
0.6
0.4
0.2
rs979037
−log10(p−value)
Recombination rate (cM/Mb)
10
8
6
4
2
0
100
80
60
40
20
0
GFOD1
MCUR1
CD83
JARID2
SIRT5
RNF182
LINC01108
JARID2−AS1
NOL7
RANBP9
13.5
14
14.5
15
Position on chr6 (Mb)

c.

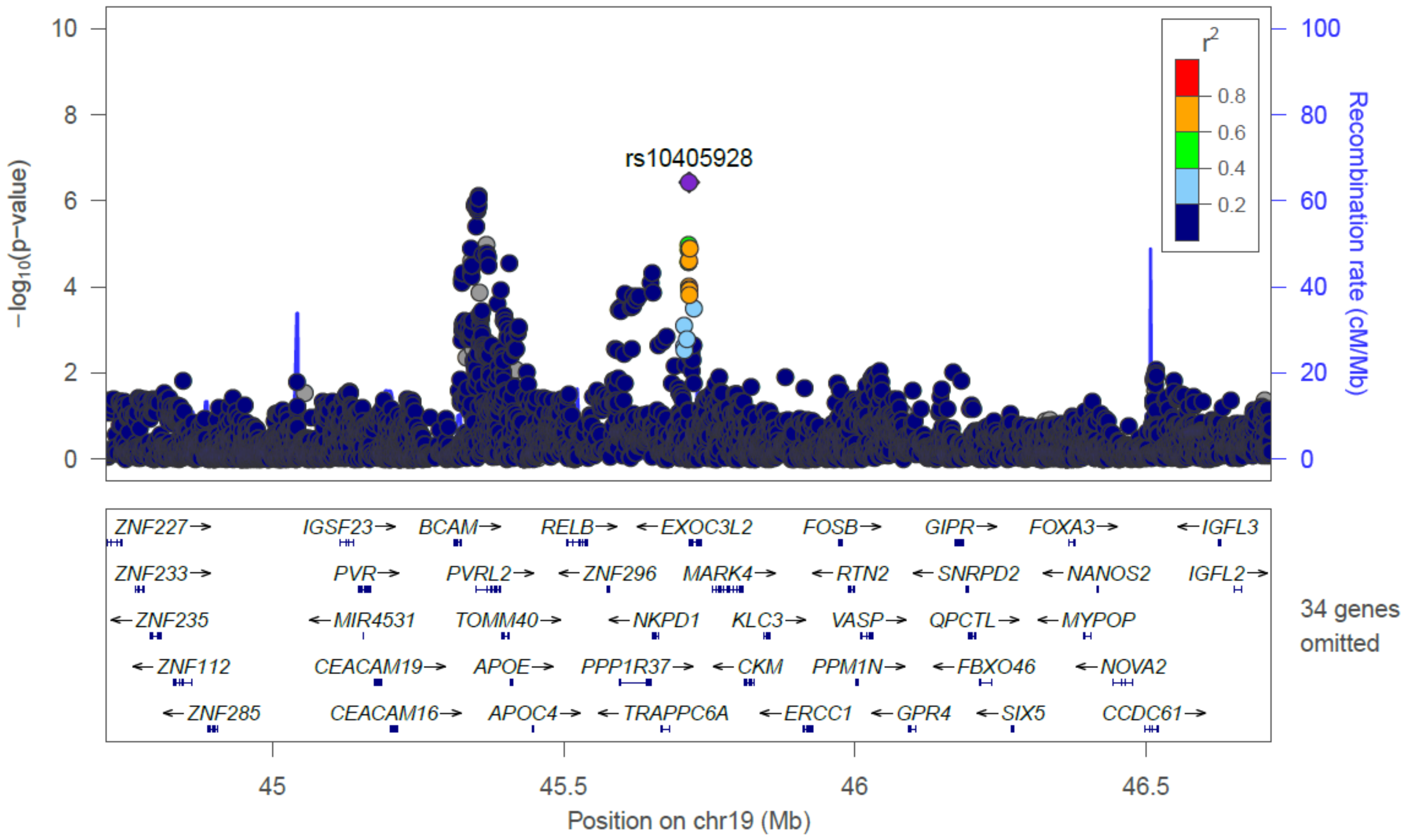

rs10405928
−log10(p-value)
Recombination rate (cM/Mb)
r2
0.8
0.6
0.4
0.2
10
8
6
4
2
0
100
80
60
40
20
ZNF227 ZNF233 ZNF235 ZNF112 ZNF285 IGSF23 PVR MIR4531 CEACAM19 CEACAM16 BCAM PVRL2 TOMM40 APOE APOC4 RELB ZNF296 NKPD1 PPP1R37 TRAPPC6A EXOC3L2 MARK4 KLC3 CKM ERCC1 FOSB RTN2 VASP PPM1N GPR4 GIPR SNRPD2 QPCTL FBXO46 SIX5 FOXA3 NANOS2 MYPOP NOVA2 CCDC61 IGFL3 IGFL2
34 genes omitted
45
45.5
46
46.5
Position on chr19 (Mb)

Supplementary Figure 7. LocusZoom plots of regions identified in the sex-specific (GERA+UKB) analyses that show differential association with cataract across women and men. The following regions were significant in women ($P<5x10^{-8}$) but not significant ($P>0.05$) in men: **a.** *GSTM2*, **b.** *DNMBP-CPN1*, and **c.** *CASP7*; the following regions were genome-wide significant in men but not in women: **d.** *QKI*, **e.** *SEMA4D*, **f.** *RBFOX1*, and **g.** *JAG1*. The y-axis represents the $-\log_{10}$(P-value); all P-values derived from logistic regression model are two-sided.

a.

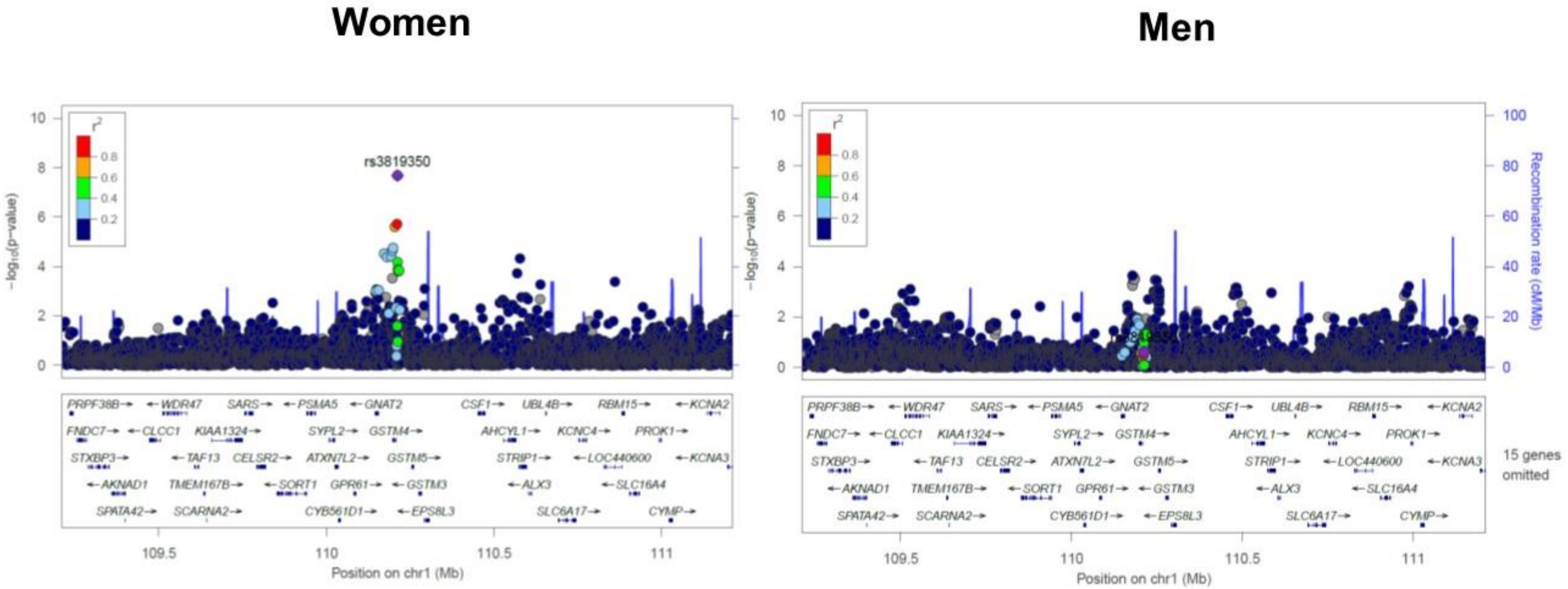

b.

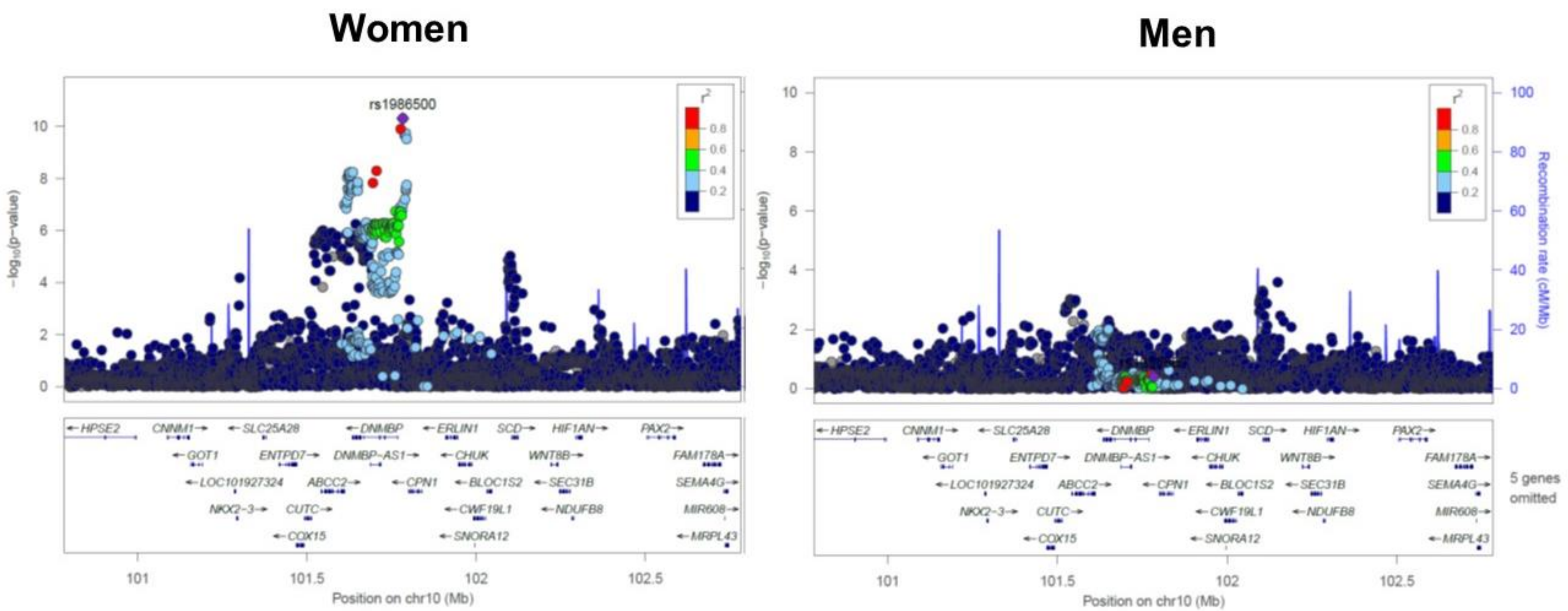
Women
Men
rs1986500
r2
−log10(p−value)
Recombination rate (cM/Mb)
HPSE2
CNNM1
SLC25A28
DNMBP
ERLIN1
SCD
HIF1AN
PAX2
GOT1
ENTPD7
DNMBP−AS1
CHUK
WNT8B
FAM178A
LOC101927324
ABCC2
CPN1
BLOC1S2
SEC31B
SEMA4G
NKX2−3
CUTC
CWF19L1
NDUFB8
MIR608
COX15
SNORA12
MRPL43
5 genes omitted
Position on chr10 (Mb)

c.

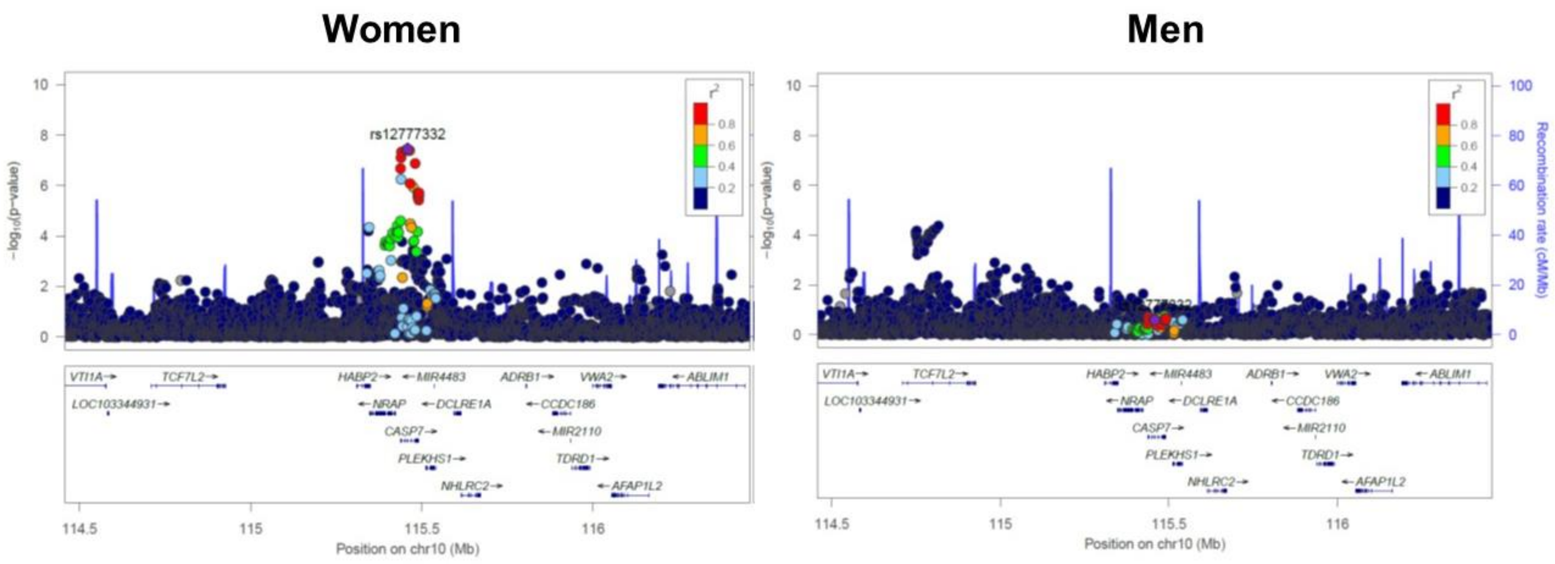
Women
Men
rs12777332
r²
0.8
0.6
0.4
0.2
−log₁₀(p-value)
Recombination rate (cM/Mb)
VTI1A→
TCF7L2→
HABP2→
←MIR4483
ADRB1→
VWA2→
←ABLIM1
LOC103344931→
←NRAP
←DCLRE1A
←CCDC186
CASP7→
←MIR2110
PLEKHS1→
TDRD1→
NHLRC2→
←AFAP1L2
114.5
115
115.5
116
Position on chr10 (Mb)

d.

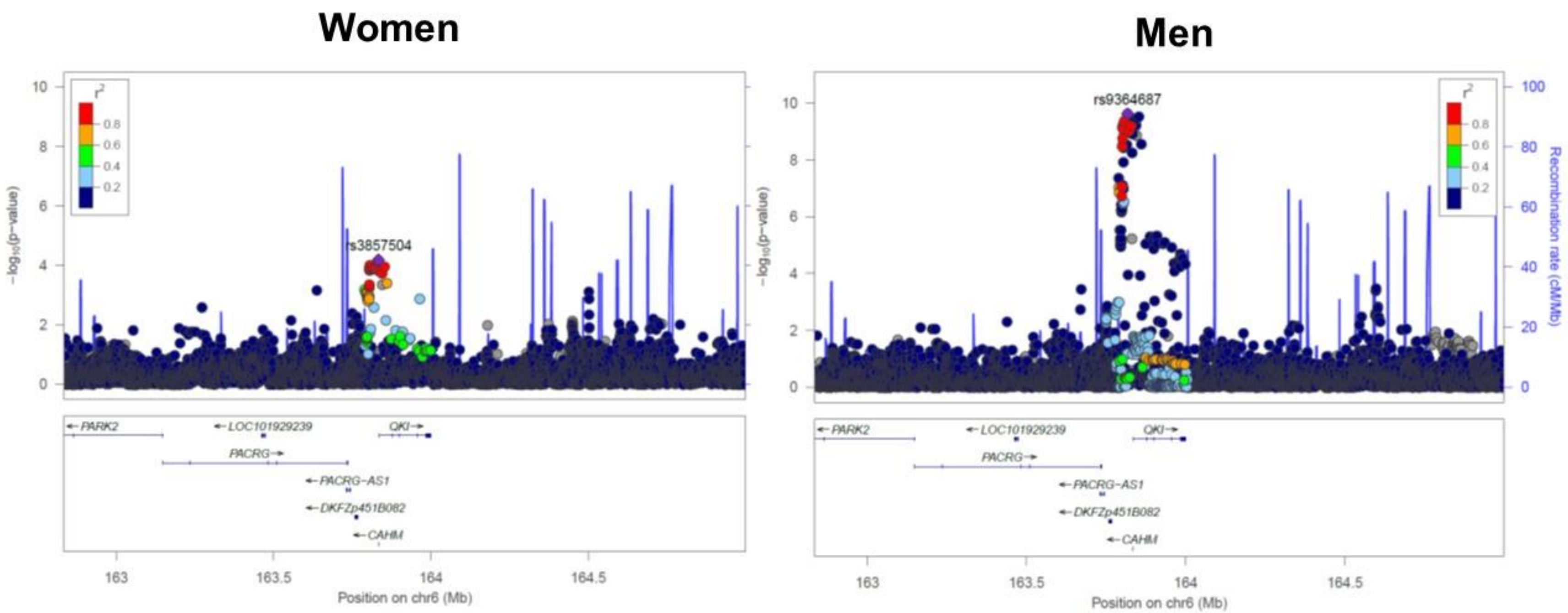
Women
Men
rs3857504
rs9364687
r²
0.8
0.6
0.4
0.2
−log10(p−value)
Recombination rate (cM/Mb)
PARK2
LOC101929239
QKI
PACRG
PACRG−AS1
DKFZp451B082
CAHM
163
163.5
164
164.5
Position on chr6 (Mb)

e.

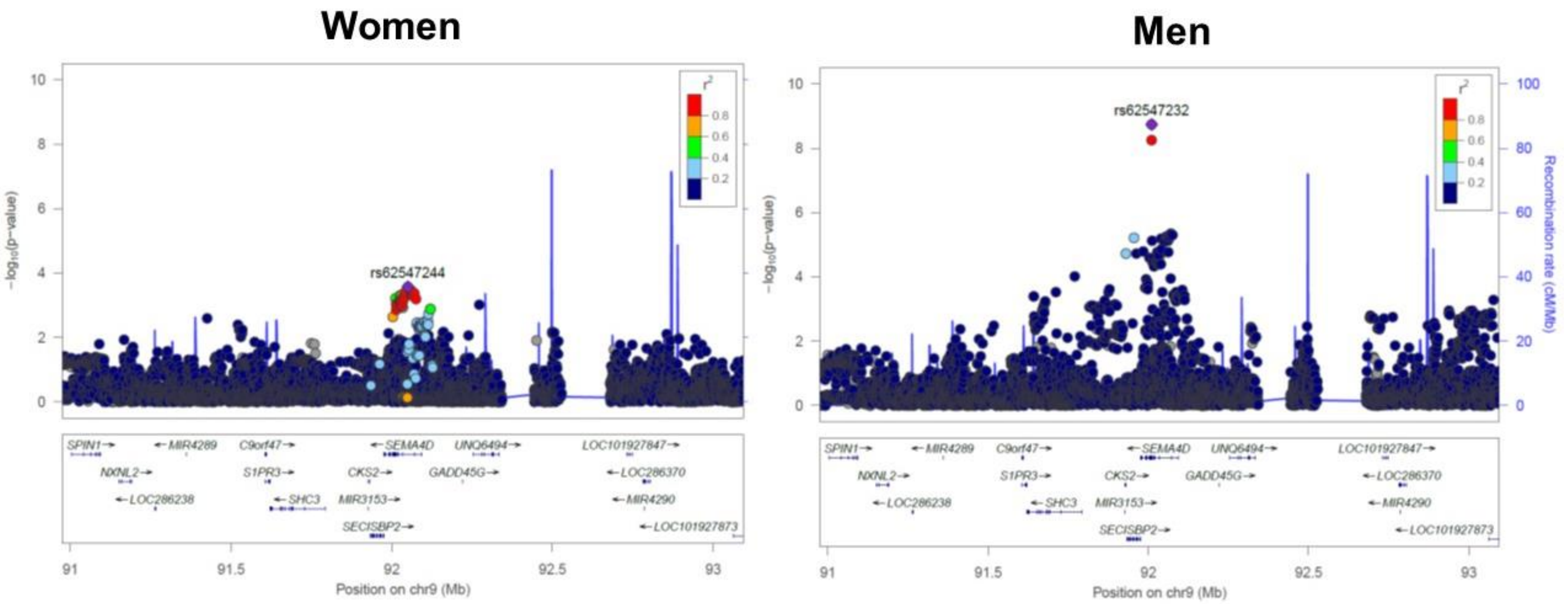

Women
Men
rs62547244
rs62547232
-log10(p-value)
Recombination rate (cM/Mb)
r2
0.8
0.6
0.4
0.2
SPIN1
MIR4289
C9orf47
SEMA4D
UNQ6494
LOC101927847
NXNL2
S1PR3
CKS2
GADD45G
LOC286370
LOC286238
SHC3
MIR3153
MIR4290
SECISBP2
LOC101927873
Position on chr9 (Mb)

f.

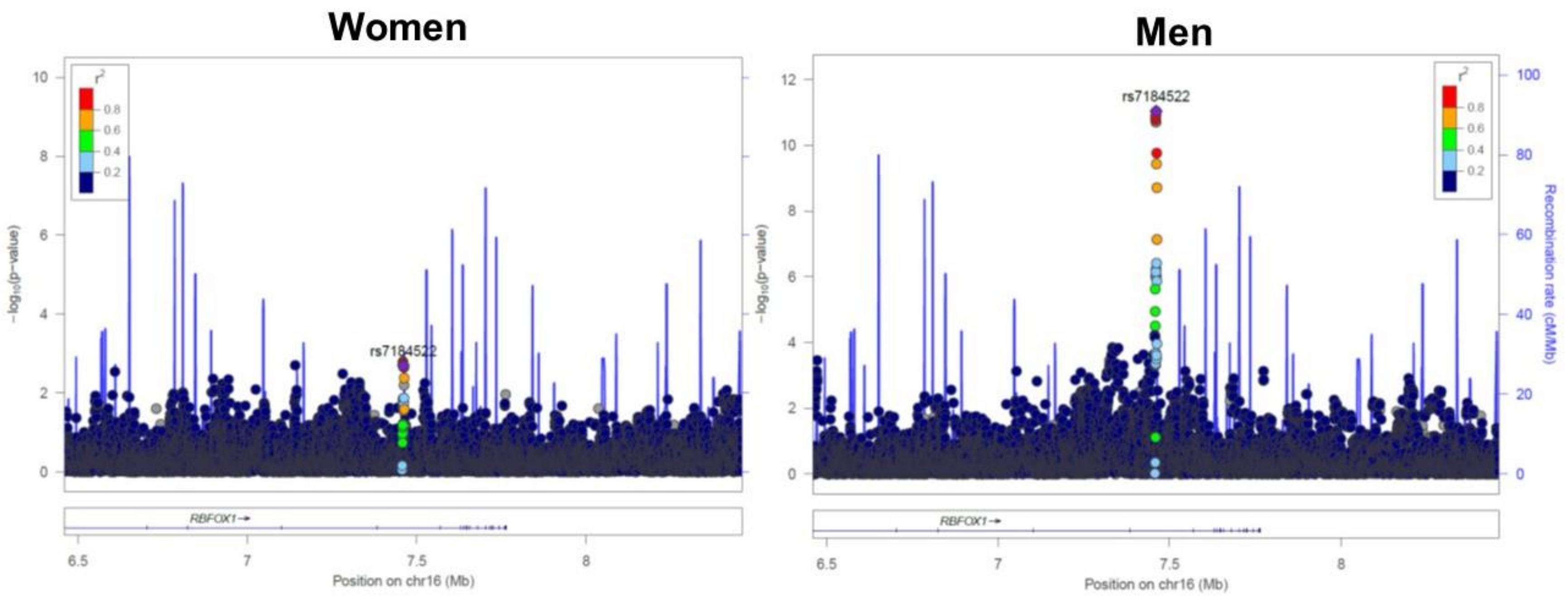

Women
Men
rs7184522
rs7184522
r²
0.8
0.6
0.4
0.2
−log10(p−value)
Recombination rate (cM/Mb)
RBFOX1→
Position on chr16 (Mb)
6.5
7
7.5
8

g.

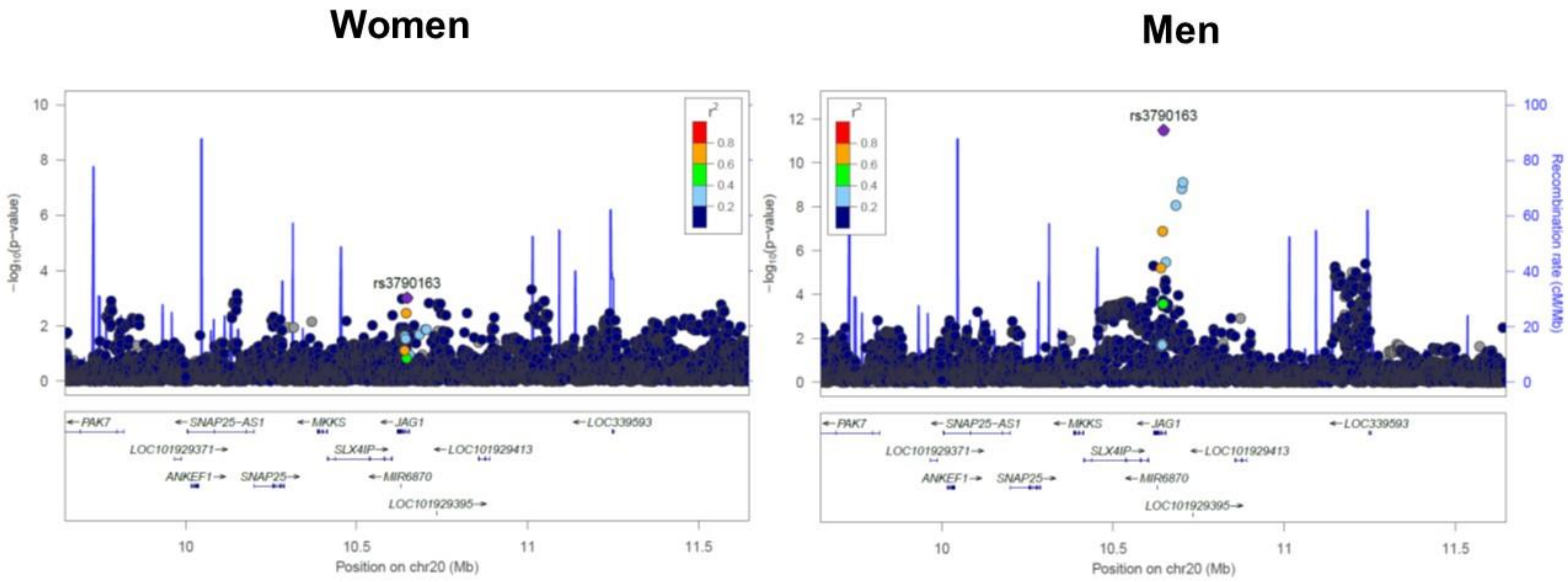

Women
Men
rs3790163
rs3790163
r2
0.8
0.6
0.4
0.2
-log10(p-value)
Recombination rate (cM/Mb)
PAK7
SNAP25-AS1
MKKS
JAG1
LOC339593
LOC101929371
SLX4IP
LOC101929413
ANKEF1
SNAP25
MIR6870
LOC101929395
Position on chr20 (Mb)

Supplementary Figure 8. Correlation of effect sizes for cataract between women and men for the lead 54 SNPs identified in the combined (GERA+UKB) GWAS multiethnic analysis and for the additional SNPs identified in the sex-specific analyses. The effect sizes were compared using a correlation test, two-sided; *P*-value for the correlation test is 5.8×10^{-8}.

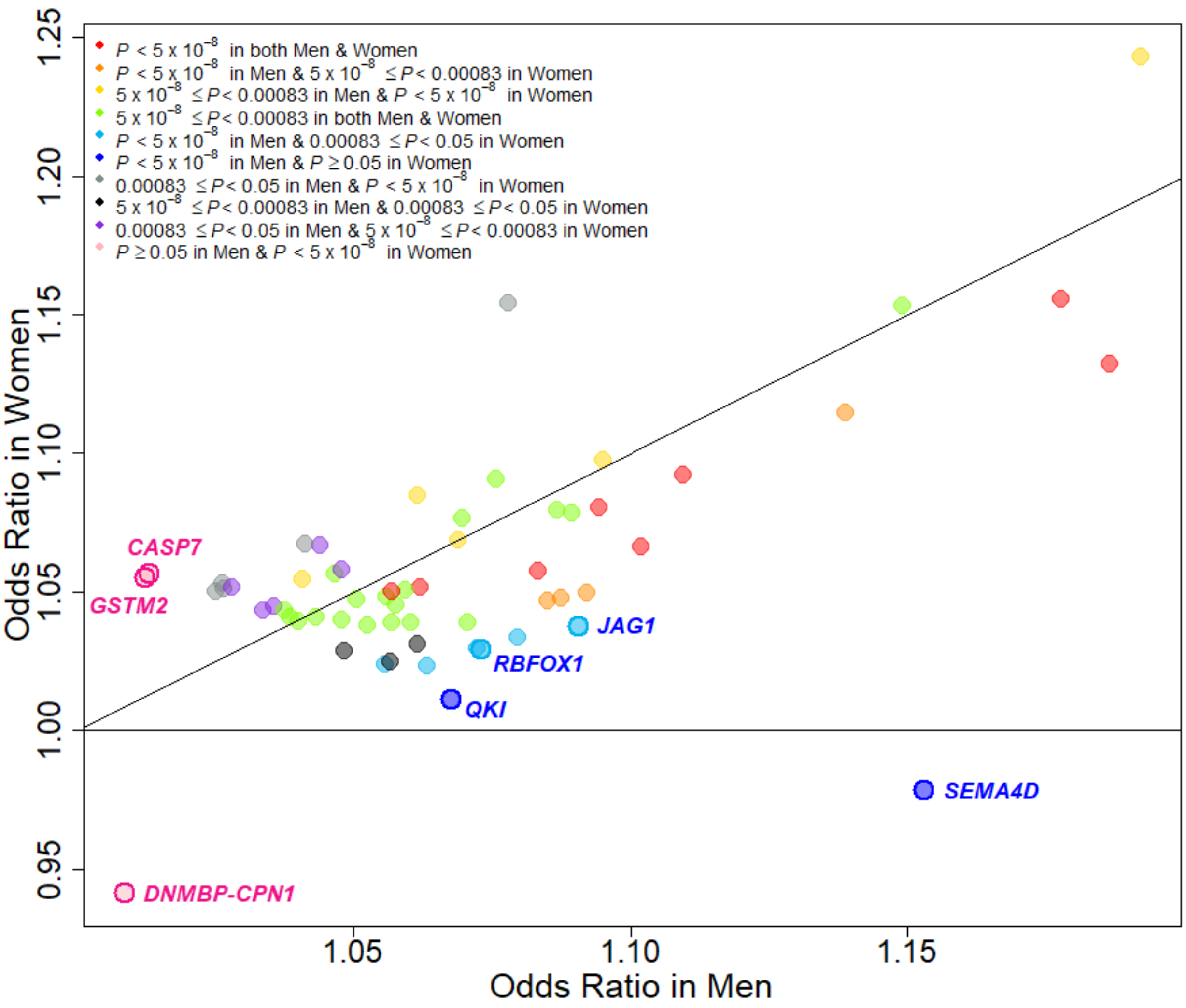

Here we report the locus names (in dark pink (or blue) color) for the lead SNPs that are specific to women (or men) and that have a significantly different OR between the women- and men-specific analyses.

Supplementary Figure 9. Enriched expression of candidate genes in mouse lens. Mouse orthologs of the human candidate genes were examined for their lens enriched-expression in the iSyTE database. “Enriched expression” in the lens is estimated by analyzing candidate gene expression in the lens compared to that in whole-embryonic body (WB). Analysis of whole lens tissue data on various platforms, microarrays (Affymetrix, Illumina) and RNA-seq indicates lens enriched expression of genes at different stages indicated by embryonic (E) and postnatal (P) days and ranged from early lens development (*i.e.* E10.5) through adulthood (*i.e.* P60). Note: P28 in Affymetrix represents expression data on isolated lens epithelium. The range of expression on each platform is indicated by a specific heat-map. The numbers within individual tiles represent fold-change differences in the level of enriched expression in the lens compared to WB.

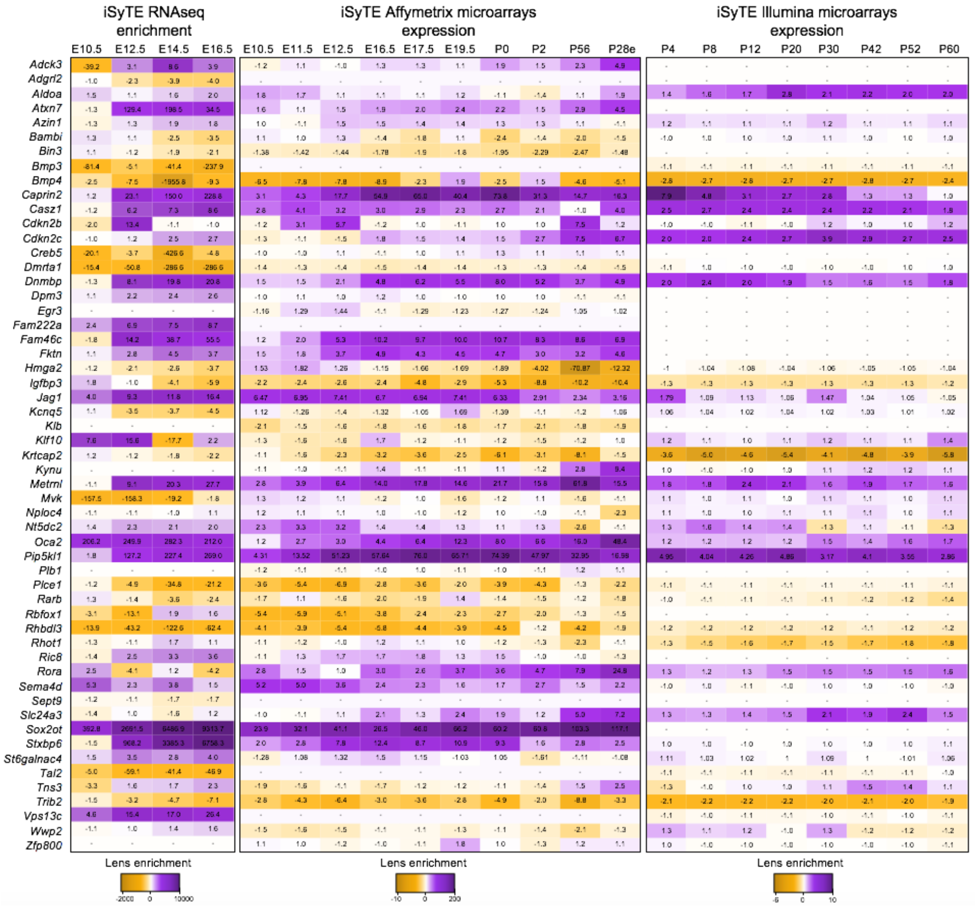

Supplementary Figure 10. Expression of candidate genes in lenses of gene perturbation mouse models with lens defects/cataract. Thirty-eight mouse orthologs of the human candidate genes in the 54 loci were examined for their expression in lens of ten different mouse models that exhibit lens defects and/or cataract. Analysis on the Affymetrix and Illumina platforms indicate expression of genes in the lens at different stages indicated by embryonic (E) and postnatal (P) days and ranged from mid lens development (*i.e.* E15.5) through adulthood (*i.e.* P56). Note: P28 in Affymetrix represents expression data on isolated lens epithelium. For individual candidate genes, the graphical representation is given. The *y*-axis indicates gene expression changes in fold-change (*$P \leq 0.05$, **$P \leq 0.01$, ***$P \leq 0.001$) in the gene-perturbation mouse model lens compared to control and the *x*-axis indicates the different mouse models and their age. The statistical analysis used was limma using lmFit and makeContrasts functions. Microarray datasets were obtained from Gene Expression Omnibus (GEO) database. Details on the GEO accession number and the number of biological replicates for specific gene-perturbation models is provided in the Methods section.

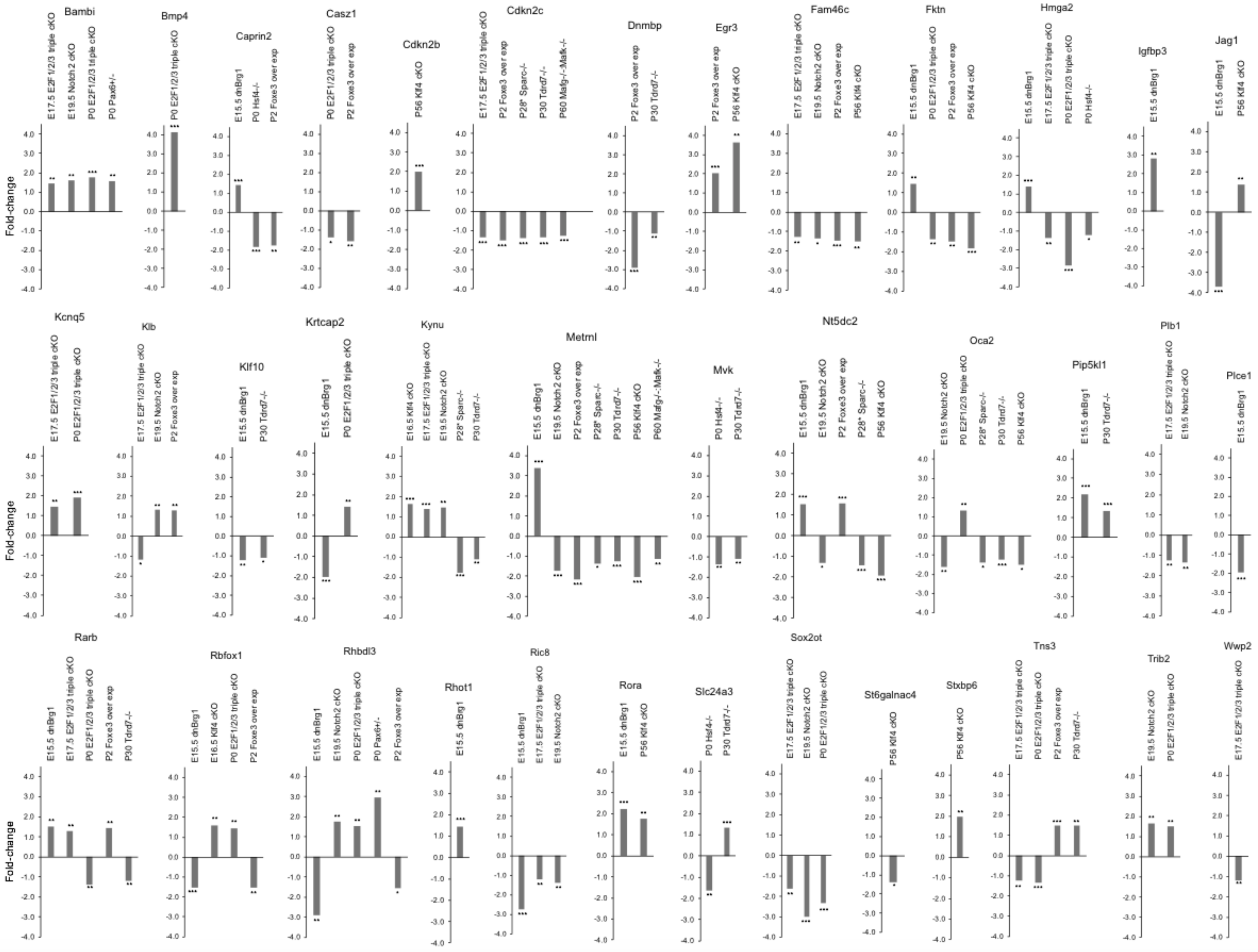
Fold-change
Bambi
E17.5 E2F1/2/3 triple cKO
E19.5 Notch2 cKO
P0 E2F1/2/3 triple cKO
P0 Pax6+/-
Bmp4
P0 E2F1/2/3 triple cKO
Caprin2
E15.5 dnBrg1
P0 Hsf4-/-
P2 Foxe3 over exp
Casz1
P0 E2F1/2/3 triple cKO
P2 Foxe3 over exp
Cdkn2b
P56 Klf4 cKO
Cdkn2c
E17.5 E2F1/2/3 triple cKO
P2 Foxe3 over exp
P28* Sparc-/-
P30 Tdrd7-/-
P60 Mafg-/-:Mafk-/-
Dnmbp
P2 Foxe3 over exp
P30 Tdrd7-/-
Egr3
P2 Foxe3 over exp
P56 Klf4 cKO
Fam46c
E17.5 E2F1/2/3 triple cKO
E19.5 Notch2 cKO
P2 Foxe3 over exp
P56 Klf4 cKO
Fktn
E15.5 dnBrg1
P0 E2F1/2/3 triple cKO
P2 Foxe3 over exp
P56 Klf4 cKO
Hmga2
E15.5 dnBrg1
E17.5 E2F1/2/3 triple cKO
P0 E2F1/2/3 triple cKO
P0 Hsf4-/-
Igfbp3
E15.5 dnBrg1
Jag1
E15.5 dnBrg1
P56 Klf4 cKO
Kcnq5
E17.5 E2F1/2/3 triple cKO
P0 E2F1/2/3 triple cKO
Klb
E17.5 E2F1/2/3 triple cKO
E19.5 Notch2 cKO
P2 Foxe3 over exp
Klf10
E15.5 dnBrg1
P30 Tdrd7-/-
Krtcap2
E15.5 dnBrg1
P0 E2F1/2/3 triple cKO
Kynu
E16.5 Klf4 cKO
E17.5 E2F1/2/3 triple cKO
E19.5 Notch2 cKO
P28* Sparc-/-
P30 Tdrd7-/-
Metrnl
E15.5 dnBrg1
E19.5 Notch2 cKO
P2 Foxe3 over exp
P28* Sparc-/-
P30 Tdrd7-/-
P56 Klf4 cKO
P60 Mafg-/-:Mafk-/-
Mvk
P0 Hsf4-/-
P30 Tdrd7-/-
Nt5dc2
E15.5 dnBrg1
E19.5 Notch2 cKO
P2 Foxe3 over exp
P28* Sparc-/-
P56 Klf4 cKO
Oca2
E19.5 Notch2 cKO
P0 E2F1/2/3 triple cKO
P28* Sparc-/-
P30 Tdrd7-/-
P56 Klf4 cKO
Pip5kl1
E15.5 dnBrg1
P30 Tdrd7-/-
Plb1
E17.5 E2F1/2/3 triple cKO
E19.5 Notch2 cKO
Plce1
E15.5 dnBrg1
Rarb
E15.5 dnBrg1
E17.5 E2F1/2/3 triple cKO
P0 E2F1/2/3 triple cKO
P2 Foxe3 over exp
P30 Tdrd7-/-
Rbfox1
E15.5 dnBrg1
E16.5 Klf4 cKO
P0 E2F1/2/3 triple cKO
P2 Foxe3 over exp
Rhbdl3
E15.5 dnBrg1
E19.5 Notch2 cKO
P0 E2F1/2/3 triple cKO
P0 Pax6+/-
P2 Foxe3 over exp
Rhot1
E15.5 dnBrg1
Ric8
E15.5 dnBrg1
E17.5 E2F1/2/3 triple cKO
E19.5 Notch2 cKO
Rora
E15.5 dnBrg1
P56 Klf4 cKO
Slc24a3
P0 Hsf4-/-
P30 Tdrd7-/-
Sox2ot
E17.5 E2F1/2/3 triple cKO
E19.5 Notch2 cKO
P0 E2F1/2/3 triple cKO
St6galnac4
P56 Klf4 cKO
Stxbp6
P56 Klf4 cKO
Tns3
E17.5 E2F1/2/3 triple cKO
P0 E2F1/2/3 triple cKO
P2 Foxe3 over exp
P30 Tdrd7-/-
Trib2
E19.5 Notch2 cKO
P0 E2F1/2/3 triple cKO
Wwp2
E17.5 E2F1/2/3 triple cKO

Supplementary Figure 11. RT-PCR based validation of candidate gene expression in the lens. Several GWAS-identified candidates were independently validated by reverse transcriptase (RT)-polymerase chain reaction (PCR) assay for their expression in mouse lens at embryonic day (E)16.5 and postnatal day (P)3. Gel images are representative of RT-PCR assays performed in three biological replicates. The specific *Actb* loading control assigned for the samples below was run on the same gel.

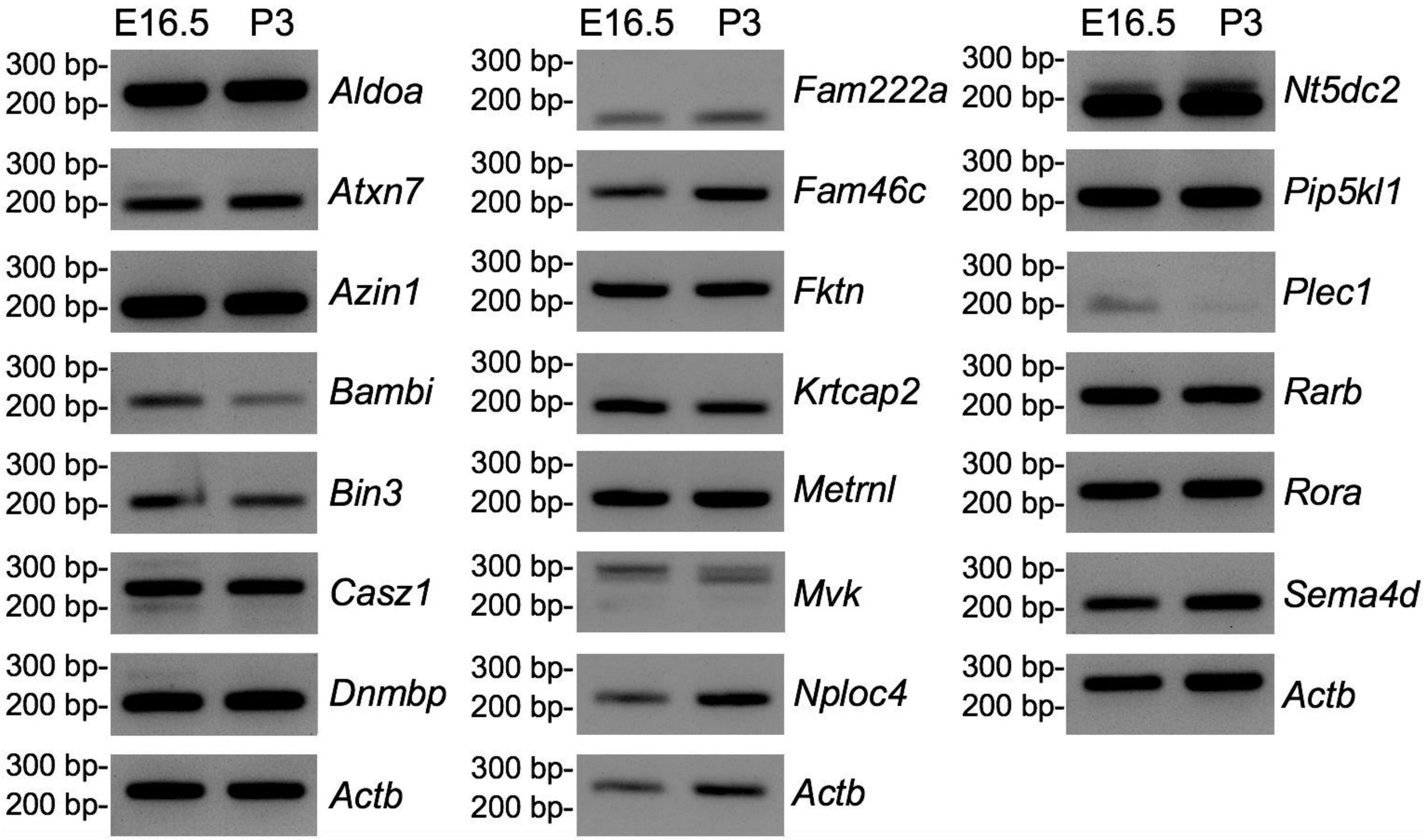

Supplementary Figure 11. RT-PCR based validation of candidate gene expression in the lens. Original full gel scans.

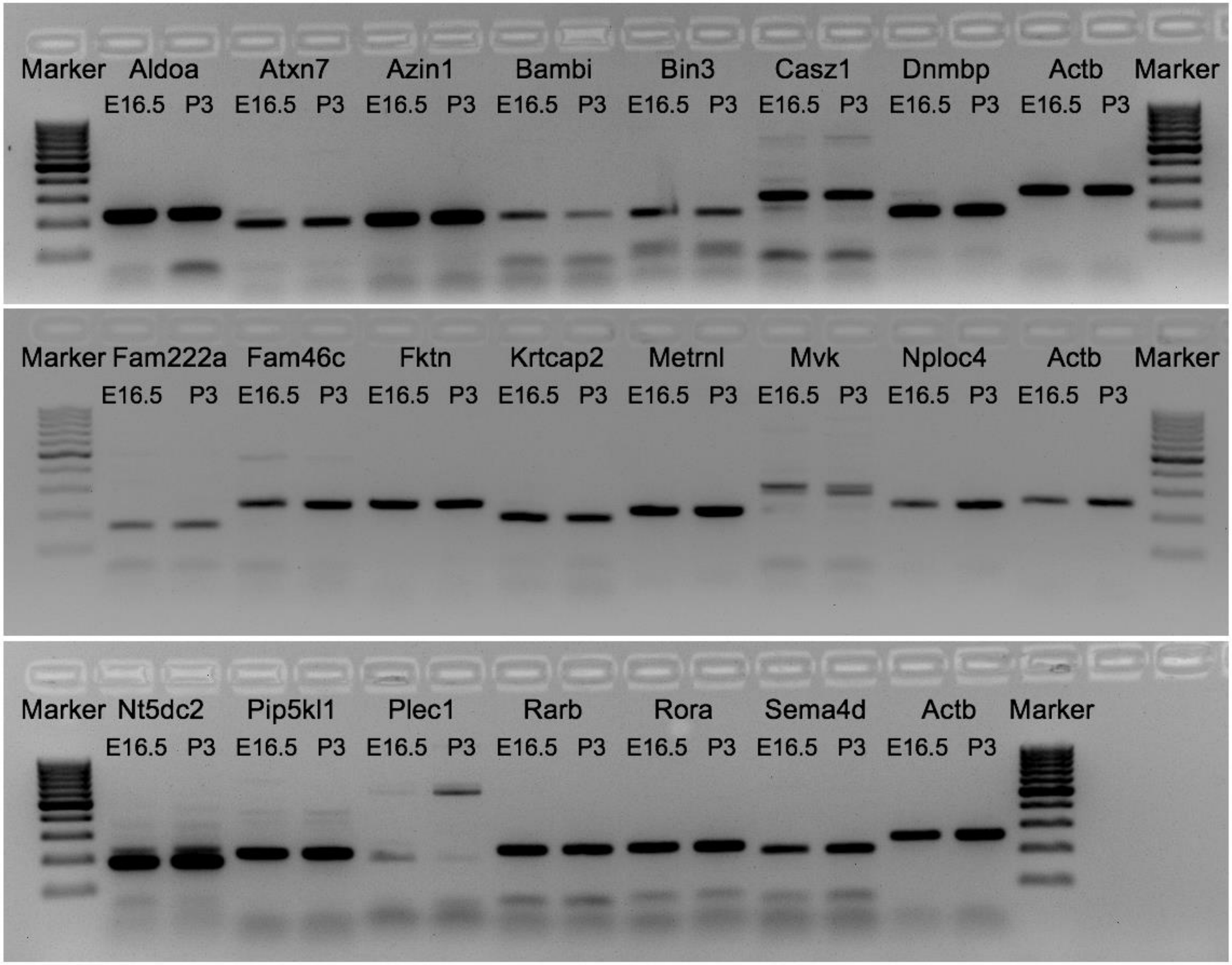

Supplement: Supplementary file 1 — Supplementary Information [file 41467_2021_23873_MOESM1_ESM.pdf]
