# Supplementary material for: A large multiethnic GWAS meta-analysis of cataract identifies new risk loci and sex-specific effects
Source: Nat Commun. 2021 Jun 14;12:3595. doi: 10.1038/s41467-021-23873-8 (PMC8203611; doi:10.1038/s41467-021-23873-8)
Supplement: Supplementary file 3 — Description of Additional Supplementary Files [file 41467_2021_23873_MOESM3_ESM.pdf]

Description of Additional Supplementary Files

File Name: Supplementary Data 1
Description: **Characteristics of cataract cases and controls from GERA, UK Biobank cohort, and 23andMe.**

File Name: Supplementary Data 2
Description: **Replication of previous cataract GWAS results (Boutin et al. Hum Mol Genet. 2020) in the GERA cohort.** All odds ratio (OR) and P-values derived from logistic regression model are two-sided. As 19 SNPs (available) were tested, the P-value adjusted for Bonferroni correction was set as P<0.00263 (0.05/19).

File Name: Supplementary Data 3
Description: **Additional cataract-associated loci identified in the European-specific meta-analysis (GERA non-Hispanic whites + UKB Europeans) and look-up in the other ethnic groups.** All odds ratio (OR) and P-values derived from logistic regression model are two-sided.

File Name: Supplementary Data 4
Description: **Cataract-associated loci identified in the European-specific metaanalysis (GERA non-Hispanic whites + UKB Europeans).** All odds ratio (OR) and P-values derived from logistic regression model are two-sided.

File Name: Supplementary Data 5
Description: **Cataract-associated variants identified in the conditional and joint multiple-SNP (COJO) analysis conducted on the European-specific (GERA non-Hispanic whites + UKB Europeans) meta-analysis results.** In grey are already known cataract-associated loci. Linkage disequilibrium (LD) metrics (R2 and D') have all been calculated in European-ancestry populations using a web-based bioinformatic tool (https://analysistools.nci.nih.gov/LDlink/). Abbreviations: P, P-value from COJO analyses; aindex SNP associated with cataract from COJO analyses; bSNPs associated with cataract in a previous study.

File Name: Supplementary Data 6
Description: **Cataract-associated loci identified in the women sex-specific combined (GERA+UKB) meta-analysis and look-up in men (GERA+UKB).** The beta coefficients from the 2 regressions were compared using a Z test (two-sided). As 20 SNPs were tested, the P-value adjusted for Bonferroni correction was set as P<0.0025 (0.05/20); while P<0.0025 are highlighted in red, P<0.05 are highlighted in pink. Loci indicated in bold are additional novel.

File Name: Supplementary Data 7
Description: **Cataract-associated loci identified in the men sex-specific combined (GERA+UKB) meta-analysis and look-up in women (GERA+UKB). The beta coefficients from the 2 regressions were compared using a Z test (two-sided).** As 21 SNPs were tested, the P-value adjusted for Bonferroni correction was set as P<0.0024 (0.05/21); while P<0.0024 are highlighted in red, P<0.05 are highlighted in pink.

File Name: Supplementary Data 8
Description: **List of the 95% credible set of variants in each of the 54 cataract loci identified in the combined (GERA+UKB) multiethnic analysis.**

File Name: Supplementary Data 9
Description: **VEGAS2 Gene-based association analysis.** For each gene definition, the n SNPs' p-values are first converted to upper tail chi-squared (χ2) statistics with one degree of freedom (df) and then summed to calculate a gene-based test statistic that would have a χ2 distribution with n df under the null hypothesis, if SNPs are in linkage equilibrium. Significance is computed by comparing the summed χ2 statistics for each gene to simulated replicates from a multivariate normal distribution with mean = 0 and variance = ∑ (Mishra, A., & Macgregor, S. Twin Research and Human Genetics 2015). Finally, the combined p-value was calculated with Fisher's method. Entries were sorted by significance (P-value) and only entries above a nominal significance cutoff (P-value<0.05) are shown in this table. As 22,673 genes were tested, the Pvalue adjusted for Bonferroni correction was set as P<2.21x10-6 (0.05/22,673); genes that reached this Bonferroni-level of significance are highlighted in pink.

File Name: Supplementary Data 10
Description: **Expression values of candidate genes in lenses of gene perturbation mouse models with lens defects/cataract**. Expression of mouse orthologs of the candidate genes was examined in the context of ten different gene perturbation conditions in transgenic, mutant, or targeted knockout mouse models that exhibit lens defects and/or cataract. Comparative analysis was performed in limma using lmFit and makeContrasts function to identify differential expression, which is provided in fold-change along with P-values. The data used to generate the bar-graph in supplementary figure 10 are highlighted.

File Name: Supplementary Data 11
Description: **VEGAS2-Pathways association analysis top results.** Entries were sorted by significance (P-value) and only entries above a nominal significance cutoff (P-value<0.05) are shown in this table. As 9,732 pathways or gene-sets from the Biosystem's database were tested, the P-value adjusted for Bonferroni correction was set as P<5.14x10-6 (0.05/9,732); the unique pathway/gene-set that reached this Bonferroni-level of significance is highlighted in pink.

File Name: Supplementary Data 12
Description: **Genetic risk sharing between cataract and other diseases/traits.** The results shown are those from an LD Score regression of the cataract meta-analysis (combining GERA non-Hispanic whites and UKB Europeans) and publicly available GWAS summary statistics (for N=772 diseases/traits at the time of writing) (Brendan Bulik-Sullivan et al. Nat Genet. 2015). All the GWAS publicly available used to establish this table were conducted in European populations. Entries were sorted by significance (P-value) and only entries above a nominal significance cutoff (P-value<0.05) are shown in this table. The traits that reached the Bonferroni-level of significance are highlighted in light orange.

File Name: Supplementary Data 13
Description: **PheWAS Results (UKB European data using GeneATLAS).** Complex traits and disorders were combined in 6 categories: in green color, disorders of lens; in blue, metabolic/co-morbidities; in yellow, anthropometric traits; in pink, blood cells related traits; in purple, venous related diseases; in grey, skin related traits.

File Name: Supplementary Data 14
Description: **Potential candidate genes within the 54 cataract-associated loci identified and relevance from previous works.** Highlighted in grey are previously reported loci (Boutin et al. HMG 2020)

File Name: Supplementary Data 15

Description: **List of primers used for RT-PCR analysis in mouse lens**
