# Supplementary material for: A large multiethnic GWAS meta-analysis of cataract identifies new risk loci and sex-specific effects
Source: Nat Commun. 2021 Jun 14;12:3595. doi: 10.1038/s41467-021-23873-8 (PMC8203611; doi:10.1038/s41467-021-23873-8)
Supplement: Supplementary file 5 — Reporting Summary [file 41467_2021_23873_MOESM5_ESM.pdf]

nature research

Corresponding author(s): Hélène Choquet

Last updated by author(s): Apr 13, 2021

Reporting Summary

Nature Research wishes to improve the reproducibility of the work that we publish. This form provides structure for consistency and transparency in reporting. For further information on Nature Research policies, see our Editorial Policies and the Editorial Policy Checklist.

Statistics

For all statistical analyses, confirm that the following items are present in the figure legend, table legend, main text, or Methods section.

n/a	Confirmed	
☐	☒	The exact sample size (*n*) for each experimental group/condition, given as a discrete number and unit of measurement
☐	☒	A statement on whether measurements were taken from distinct samples or whether the same sample was measured repeatedly
☐	☒	The statistical test(s) used AND whether they are one- or two-sided<br>*Only common tests should be described solely by name; describe more complex techniques in the Methods section.*
☐	☒	A description of all covariates tested
☐	☒	A description of any assumptions or corrections, such as tests of normality and adjustment for multiple comparisons
☐	☒	A full description of the statistical parameters including central tendency (e.g. means) or other basic estimates (e.g. regression coefficient) AND variation (e.g. standard deviation) or associated estimates of uncertainty (e.g. confidence intervals)
☐	☒	For null hypothesis testing, the test statistic (e.g. *F*, *t*, *r*) with confidence intervals, effect sizes, degrees of freedom and *P* value noted<br>*Give P values as exact values whenever suitable.*
☒	☐	For Bayesian analysis, information on the choice of priors and Markov chain Monte Carlo settings
☐	☒	For hierarchical and complex designs, identification of the appropriate level for tests and full reporting of outcomes
☐	☒	Estimates of effect sizes (e.g. Cohen's *d*, Pearson's *r*), indicating how they were calculated

Our web collection on statistics for biologists contains articles on many of the points above.

Software and code

Policy information about availability of computer code

Data collection	Genotype analysis, quality control, phasing, and imputation analysis were performed on samples from the GERA cohort using the following softwares: Genotyping Console™ Software (Affymetrix) v4.0 to perform genotype calling, quality control (QC) analysis, and sample or SNP filtering prior to downstream analysis. PLINK software v1.90 to perform additional QC analyses. Genotypes were then pre-phased with Eagle5 v2.3.2, and then imputed with Minimac36 v2.0.1. For the iSyTE analyses for lens gene expression, microarray files were imported in the R statistical environment (http://www.r-project.org), and processed using relevant packages implemented in Bioconductor v3.12 (https://www.bioconductor.org). All software programs employed are available for public use and no custom code was employed.
Data analysis	Eigenstrat v4.2 was used to calculate the principal components (PCs) on each of the four GERA ethnic groups. PLINK v1.9 was used to perform a logistic regression of the outcome and each SNP. Other statistic analyses and data management were performed in the language-and-environment R, version 3.6.0, using functions from the default libraries. Genome-wide Complex Trait Analysis (GCTA) integrative tool was used to conduct a multi-SNP-based conditional & joint association analysis (COJO). CAVIARBF was used to prioritize genetic variants within the identified genomic regions. The Versatile Gene-based Association Study - 2 version 2 (VEGAS2v02) web platform was used to prioritize genes and biological pathways. The iSyTE database was used to analyze mouse orthologs of the human candidate genes. The iSyTE database was also used to examine expression of mouse orthologs of the candidate genes in the context of nine different gene perturbation conditions in transgenic, mutant, or targeted knockout mouse models that exhibit lens defects and/or cataract. The LD Hub web interface was used to estimate the genetic correlation of cataract with more than 700 diseases/traits. The Roslin Gene Atlas was used to conduct PheWAS analyses.

For manuscripts utilizing custom algorithms or software that are central to the research but not yet described in published literature, software must be made available to editors and reviewers. We strongly encourage code deposition in a community repository (e.g. GitHub). See the Nature Research guidelines for submitting code & software for further information.

Data

Policy information about availability of data

All manuscripts must include a data availability statement. This statement should provide the following information, where applicable:

- Accession codes, unique identifiers, or web links for publicly available datasets
- A list of figures that have associated raw data
- A description of any restrictions on data availability

The GERA genotype data are available upon application to the KP Research Bank (https://researchbank.kaiserpermanente.org/). The combined (GERA+UKB) meta-analysis GWAS summary statistics are available from the NHGRI-EBI GWAS Catalog (https://www.ebi.ac.uk/gwas/downloads/summary-statistics), study accession number GCST90014268. The variant-level data for the 23andMe replication dataset are fully disclosed in the manuscript. Individual-level data are not publicly available due participant confidentiality, and in accordance with the IRB-approved protocol under which the study was conducted. Expression or lens-enriched expression heat-map for candidate genes can be accessed through the iSyTE web-tool (https://research.bioinformatics.udel.edu/iSyTE). Pathways or gene-sets were derived from the Biosystem's database which can be accessed through the following link (https://vegas2.qimrberghofer.edu.au/biosystems20160324.vegas2pathSYM).

Field-specific reporting

Please select the one below that is the best fit for your research. If you are not sure, read the appropriate sections before making your selection.

☒ Life sciences ☐ Behavioural & social sciences ☐ Ecological, evolutionary & environmental sciences

For a reference copy of the document with all sections, see nature.com/documents/nr-reporting-summary-flat.pdf

Life sciences study design

All studies must disclose on these points even when the disclosure is negative.

Sample size

In this study, we conducted a genome-wide association analyses, followed by meta-analysis, including 585,243 individuals (67,844 cases and 517,399 cataract-free controls) from two cohorts: the Genetic Epidemiology Research in Adult Health and Aging (GERA) and the UK Biobank (UKB). Rather than performing a power calculation, we collected the largest possible GWAS for cataract to date to identify novel risk loci.

In GERA, patients with pseudophakia were diagnosed by a Kaiser Permanente ophthalmologist and were identified in the KPNC electronic health record system based on the following International Classification of Disease, Ninth (ICD9) or Tenth Revision (ICD10) diagnosis codes: V43.1 (ICD-9 code) and Z96.1 (ICD-10 code). Cataract cases were also identified if they had a history of having a cataract surgery at KPNC. Our control group included all the non-cases. In total, 33,145 patients who have undergone cataract surgery and 64,777 controls from GERA were included in this study.

In UKB, cataract cases (N=34,699) were defined as participants with a self-reported cataract operation (f20004 code 1435) or/and a hospital record including a diagnosis code (ICD-10: H25 or H26). Controls (N=452,622) were participants who were not cases.

Data exclusions

In GERA, genotype quality control (QC) procedures and imputation were conducted on an array-wise basis, after an updated genotyping algorithm with an advanced normalization step specifically for SNPs in batches not recommended or flagged by the outlier plate detector than has previously been done. Subsequently, variants were excluded if: >3 clusters were identified; the number of batches was <38/42 (EUR array), <3/5 (AFR), <3/6 (EAS), or <7/9 (LAT); and the ratio of expected allele frequency variance across packages was <100 (EUR), <50 (AFR), <100 (EAS), <200 (LAT). On the EUR array, variants were additionally excluded if heterozygosity >.52 or <.02, and if an association test between Reagent kit v1.0 and v2.0 had P<10-4. Imputation was done by array, and we additionally removed variants with call rates <90%.

Replication

Replication analyses of the loci identified in the combined (GERA+UKB) meta-analysis as well as the loci identified through the sex-specific analyses were conducted using self-reported data from a GWAS including 347,209 self-reported cataract cases and 2,887,246 controls from 23andMe, Inc., research cohort.

In 23andMe research cohort, 45 out of 51 lead SNPs available (88.2%) replicated with a consistent direction of effect at a Bonferroni corrected significance threshold of 9.8x10-4 (P-value=0.05/51) and additional 2 SNPs were nominally significant (P<0.05).

Randomization

Samples were not randomized. This is a case-control study where cases were those with cataract, and controls were non-cases. Association analyses were adjusted for age, sex, and principal components as covariates.

In GERA, patients with pseudophakia were diagnosed by a Kaiser Permanente ophthalmologist and were identified in the KPNC electronic health record system based on the following International Classification of Disease, Ninth (ICD9) or Tenth Revision (ICD10) diagnosis codes: V43.1 (ICD-9 code) and Z96.1 (ICD-10 code). Cataract cases were also identified if they had a history of having a cataract surgery at KPNC. Our control group included all the non-cases. In total, 33,145 patients who have undergone cataract surgery and 64,777 controls from GERA were included in this study.

In UKB, cataract cases (N=34,699) were defined as participants with a self-reported cataract operation (f20004 code 1435) or/and a hospital record including a diagnosis code (ICD-10: H25 or H26). Controls (N=452,622) were participants who were not cases.

In 23andMe replication analysis, cases were defined as those individuals that reported having cataract whereas controls were defined as individuals that reported not having cataract. Individuals that preferred not to/did not answer the cataract questions were excluded from the analysis.

Blinding

Blinding was not relevant to our study, since participants were selected based on their cataract status.

Reporting for specific materials, systems and methods

We require information from authors about some types of materials, experimental systems and methods used in many studies. Here, indicate whether each material, system or method listed is relevant to your study. If you are not sure if a list item applies to your research, read the appropriate section before selecting a response.

Materials & experimental systems

n/a	Involved in the study
☒	☐ Antibodies
☒	☐ Eukaryotic cell lines
☒	☐ Palaeontology and archaeology
☐	☒ Animals and other organisms
☐	☒ Human research participants
☒	☐ Clinical data
☒	☐ Dual use research of concern

Methods

n/a	Involved in the study
☒	☐ ChIP-seq
☒	☐ Flow cytometry
☒	☐ MRI-based neuroimaging

Animals and other organisms

Policy information about studies involving animals; ARRIVE guidelines recommended for reporting animal research

Laboratory animals	Mus musculus, C57BL/6J strain, age embryonic day 16.5 and postnatal day 3, before sex can be determined by visual inspection. Information on housing conditions for the mice: The light cycle is 12 hours light / 12 hours dark, the temperature is 71 Degrees F +/- 2 degrees and the humidity range is 30 – 70%.
Wild animals	The study did not involve wild type animals
Field-collected samples	The study did not involve samples collected from the field.
Ethics oversight	The University of Delaware Institutional Animal Care and Use Committee (IACUC) approved the study protocol on animals.

Note that full information on the approval of the study protocol must also be provided in the manuscript.

Human research participants

Policy information about studies involving human research participants

Population characteristics	The Genetic Epidemiology Research in Adult Health and Aging (GERA) cohort consists of 110,266 adult men and women, 18 years and older, who are of non-Hispanic white, Hispanic/Latino, Asian or African American ethnicity.<br>The UK Biobank (UKB) is a large prospective study following the health of approximately 500,000 participants from 5 ethnic groups (European, East Asian, South Asian, African British, and mixed ancestries) resident in the UK aged between 40 and 69 years-old at the baseline recruitment visit.
Recruitment	Participants from the GERA cohort are members of the Kaiser Permanente Northern California (KPNC) integrated health care delivery system, and provided self-reported information via the Research Program on Genes, Environment, and Health (RPGEH) survey.<br>For UKB participants, demographic information and medical history were ascertained through touch-screen questionnaires. UKB participants also underwent a wide range of physical and cognitive assessments, including blood sampling.<br>Potential Recruitment biases due to self-reports: the self-reported cataract in UKB is unlikely to bias the results of this study as we observed a very high concordance between the GWAS results for cataract validated cases compared to self-reported in UKB. Further, most of our results were replicated in self-reported data from 23andMe Research Cohort.
Ethics oversight	For GERA, all study procedures were approved by the Institutional Review Board of the Kaiser Permanente Northern California Institutional Review Board. Written informed consent was obtained from all participants.<br>For UKB, the analyses presented in this paper were carried out under UK Biobank Resource project #14105.<br>For 23andMe, participants provided informed consent and participated in the research online, under a protocol approved by the external AAHRPP-accredited IRB, Ethical & Independent Review Services (E&I Review).

Note that full information on the approval of the study protocol must also be provided in the manuscript.
